# The gut microbiome and child and adolescent depression and anxiety: a systematic review and meta-analysis with youth consultation

**DOI:** 10.1017/gmb.2025.10013

**Published:** 2025-09-01

**Authors:** Susan C. Campisi, Flora Zhang, Minjoo Seo, Jessica Muha, Anett Schumacher, Isabella De Luca, Glyneva Bradley-Ridout, Kaitlyn Merriman, John Parkinson, Daphne J. Korczak

**Affiliations:** 1 Nutrition and Dietetics Program, Clinical Public Health Division, Dalla Lana School of Public Health, University of Toronto, Toronto, ON, Canada; 2 Neuroscience and Mental Health, The Hospital for Sick Children, Toronto, ON, Canada; 3 Gerstein Science Information Centre, University of Toronto, Toronto, ON, Canada; 4 Program in Molecular Medicine, Hospital for Sick Children, Toronto, ON, Canada; 5 Department of Biochemistry, University of Toronto, Toronto, ON, Canada; 6 Department of Molecular Genetics, University of Toronto, Toronto, ON, Canada; 7 Department of Psychiatry, Temerty Faculty of Medicine, University of Toronto, Toronto, ON, Canada

**Keywords:** adolescents, microbiome, depression, anxiety, review

## Abstract

Decreased gut microbial diversity is associated with greater depression symptoms in adults. Findings on the relationship between the gut microbiome and depression or anxiety in children and adolescents are mixed, and evidence syntheses are needed. Seven databases were searched for peer-reviewed studies on the gut microbiome and internalizing symptoms, depression, or anxiety, in children and adolescents (<19 years). Random-effects meta-analyses of alpha diversity indices were performed. Youth advisors validated the research findings’ relevance to their experiences and contributed to dissemination planning. Eight studies were included, representing 2,865 participants (mean age = 11.4 years, SD = 4.3). Study designs were cross-sectional (*n* = 5), longitudinal (*n* = 2), and interventional (*n* = 1). No association was found between alpha or beta diversity and internalizing problems, depression, or anxiety. Increased abundance of genera within phyla Bacillota (e.g., *Fusicatenibacter*) and Pseudomonadota (e.g., *Escherichia*), along with decreased abundance of other Bacillota genera (e.g., *Faecalibacterium*), were associated with depression and anxiety symptoms. This review identified preliminary associations between specific bacterial taxa and depression and anxiety in children and adolescents. Larger studies using comprehensive analytical approaches are needed to explore the role of the gut microbiome in the genesis and treatment of internalizing disorders.

## Introduction

Depression and anxiety are serious mental health conditions that can have long-lasting consequences, especially when the age of onset is early. Globally, over 13% of adolescents experience mental health disorders. (Keeley, 2021) The most common of these include internalizing problems, such as depression and anxiety (43%), which are characterized by anhedonia and excessive worry, respectively. (Keeley, 2021) Given the high comorbidity of anxiety and depression among young people (Al-Asadi et al., 2015; Konac et al., 2021), early detection and intervention for these internalizing disorders are essential. Addressing depression and anxiety during childhood and adolescence is critical to prevent ongoing morbidity and mortality from these conditions across the lifespan. (Scott, 2014)

Interest is growing in the role of diet and the gut microbiome as a potential treatment for mental health. (Marx et al., 2017; McGuinness et al., 2022; Dawson et al., 2024) The gut microbiome, a complex ecosystem of trillions of microorganisms residing in the gastrointestinal tract, plays a crucial role in maintaining overall health and well-being. (Petersen & Round, 2014) A diverse and balanced gut microbiome is associated with positive health outcomes. Conversely, dysbiosis, defined as an imbalance in gut microbial composition, is emerging as having a potential role in the pathogenesis of diverse diseases encompassing mental health conditions. (Petersen & Round, 2014) Dietary intake significantly influences the composition of the gut microbiome by providing essential nutrients and growth substrates for different bacterial species. (Dong & Gupta, 2019; Redondo-Useros et al., 2020; Wilson et al., 2020) Thus, dietary modifications have the potential to influence the gut microbiome and improve mental health, as various diets profoundly impact the stability, functionality, and diversity of this microbial community. (Ross et al., 2024)

The gut microbiome is thought to act on mental health via the gut–brain axis, which represents a bidirectional communication pathway connecting the gastrointestinal tract, the central nervous system, and the endocrine system. (Basiji et al., 2023) The identified gut–brain axis interactions encompass metabolic, immunological, and neurological pathways, which are hypothesized to be influenced, in part, by microbially derived metabolites (e.g., short-chain fatty acids [SCFA]). (Basiji et al., 2023) The gut microbiota plays a crucial role in this communication by influencing the production and regulation of neurotransmitters. For instance, gut microbes can influence the availability of tryptophan, a precursor to the neurotransmitter serotonin, and even directly produce neurotransmitters such as gamma-aminobutyric acid and glutamate, both of which play essential roles in mood regulation. (Basiji et al., 2023) Additionally, SCFAs, produced by gut bacteria during fibre fermentation, are thought to influence blood–brain barrier permeability and possess anti-inflammatory properties, potentially impacting various physiological processes related to mental health. Specific bacterial groups, such as *Bifidobacterium* and *Lactobacillus*, have been linked to neurotransmitter modulation, gut barrier integrity, and reduced inflammation. (Yarandi et al., 2016) These factors potentially influence the development and severity of internalizing symptoms such as depression. (Trzeciak & Herbet, 2021)

Despite the growing interest in the gut microbiome and its potential impact on mental health in children and adolescents, research on the association between the gut microbiome composition and internalizing disorders, or on the effectiveness of microbiome-targeted interventions for depression and anxiety symptoms, is limited. (Romano et al., 2023) A recent umbrella review examining the relationship between the microbiome and child and adolescent mental health disorders identified the absence of systematic reviews examining depression and anxiety, highlighting the need for greater understanding in this promising area. (Romano et al., 2023) This study addresses a critical gap by synthesizing existing evidence on the relationship between the gut microbiome and depression, anxiety, and internalizing symptoms in children and adolescents. A comprehensive synthesis is essential to clarify current understanding and guide future research and microbiome-informed strategies to improve youth mental health.

A key barrier to advancing our understanding of the gut microbiome’s role in mental health is the limited number of studies involving youth. This gap may reflect a broader issue: limited awareness or perceived relevance of microbiome research among young people, which may, in turn, reduce their willingness to participate. Given that youth engagement is essential for ensuring the feasibility, validity, and translational potential of microbiome–mental health research in this age group, targeted efforts to enhance understanding and perceived relevance are critical. Systematic reviews are increasingly incorporating best practices in youth engagement, extending beyond the traditional researcher-driven analysis of existing studies. This shift grants youth agency, allowing their lived experiences to shape research questions and ensure reviewed studies address issues most relevant to their demographic. Youth involvement can also contribute to tailoring the dissemination of findings to reach intended audiences more effectively. (Nguyen et al., 2019) Youth involvement fosters critical information evaluation skills and helps build research expertise for their future endeavours. Several recent reviews on child and adolescent mental health include a youth engagement component. (Hawke et al., 2019; Cohen Kadosh et al., 2021; Raniti et al., 2022; Hielscher et al., 2023) This collaborative approach holds promise for enriching the research process and fostering more impactful findings that directly benefit young people. Thus, the current study sought to incorporate youth perspectives on study findings and inform future research, and ensure the inclusion of dissemination strategies that are meaningful to youth.

## Methods

This systematic review was conducted in accordance with the Joanna Briggs Institute (JBI) Systematic Review guidelines and is reported following the Preferred Reporting Items for Systematic Reviews and Meta-Analyses (PRISMA) checklist. (Aromataris & Munn, 2024; Page et al., 2021) The protocol was registered with the International Prospective Register of Systematic Reviews CRD 42023476178.

Systematic searches were conducted November 6–9, 2023 in the following databases: Child Development and Adolescent Studies (EBSCOhost), Ovid MEDLINE (1946–present, including Epub ahead of print, in-process, and other non-indexed citations), Ovid EMBASE (1947–present), Ovid PsycINFO (1806–present), EBSCO CINAHL Plus with Full Text (1981–present), Scopus (Elsevier), and the Cochrane CENTRAL. The search strategies were developed by two academic health sciences librarians (KM and GBR) and peer-reviewed by an independent librarian following the Peer Review of Electronic Search Strategies for systematic review guidelines. (McGowan et al., 2016) The search strategy was translated into each database using that platform’s command language, including text words, controlled vocabulary, and subject headings when possible. Full search strategies can be found in Supplementary Table 1.

Studies were considered eligible for inclusion based on the following criteria: (a) examined a paediatric population (mean age < 19 years) – studies that included a broader age range were eligible if data were stratified and reported separately for subgroups under 19 years of age; (b) included participants with a clinical diagnosis of depression or anxiety, self-reported symptoms of depression or anxiety, or internalizing problems assessed using standardized measures; (c) quantified the association between the gut microbiome and depression, anxiety, or internalizing problems, or evaluated the effects of gut microbiome-targeted interventions (e.g., prebiotics, probiotics, and synbiotics) on these outcomes; and (d) were published in peer-reviewed journals. Studies involving participants with comorbid psychiatric disorders were included, given the frequent co-occurrence of depression and anxiety with other psychiatric conditions during adolescence. (Avenevoli et al., 2015) Studies conducted among participants with other chronic health conditions (e.g., heart disease, stroke, cancer, type 2 diabetes, and obesity) were excluded. The review employed a comprehensive search strategy, identifying studies published across all publication dates, languages, and geographic locations. Studies published in languages besides English were not considered due to resource limitations for translation.

**Figure 1.**
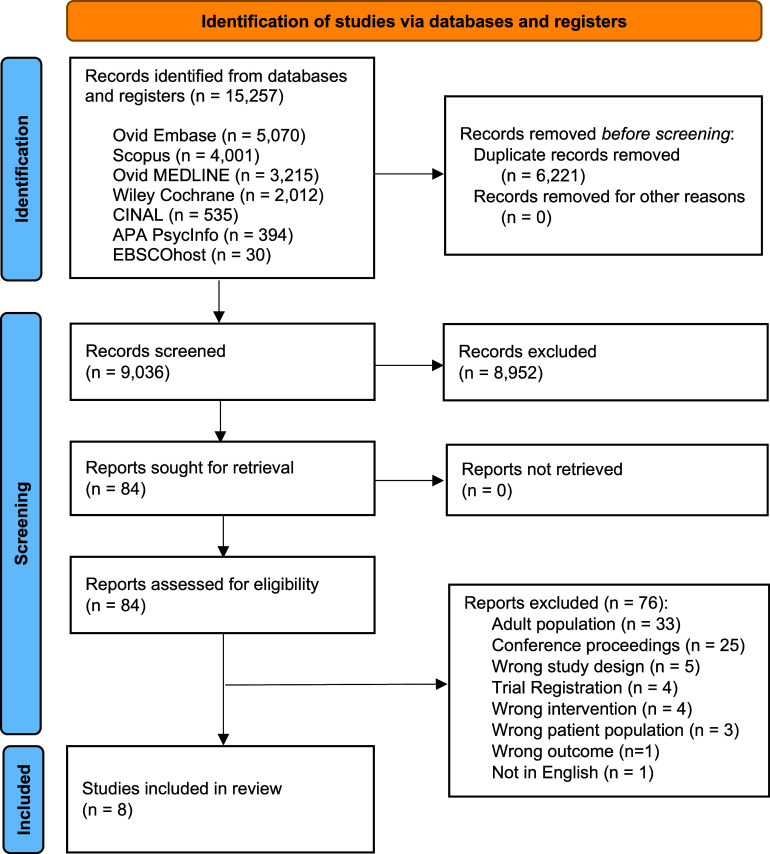
PRISMA flowchart.

Before the screening process, inclusion and exclusion criteria were discussed by the reviewers to ensure a common understanding. Deduplication was conducted with Covidence, ensuring high accuracy. (McKeown & Mir, 2021; Sandra & Mir, 2024) Study screening was completed in a two-step process, first by title and abstract, followed by full-text review. Each article was screened in duplicate independently (MS, FZ, AS, JM, and SCC) using Covidence online systematic review software with a discussion between the reviewers to achieve consensus when discrepancies arose. (Covidence Systematic Review Software, 2023) Results at all stages of screening and reasons for full-text exclusion are reported in the PRISMA Flow Diagram (Figure 1).

The data extraction template was pilot-tested on five studies and reviewed and adapted before continued extraction. Data were extracted in duplicate independently by MS and FZ, with discrepancies resolved by SCC and AS. The following data were extracted: author, year, journal, study type, sample size, mean age and range, mental health disorder, confounding variables, gut microbiota data (sequencing platform, relative abundance, richness, and alpha and beta diversity), mental health outcome data (diagnostic tool to assess mental health symptoms and symptom severity/impact) and, if relevant, type of intervention and the intervention details (duration of intervention, frequency, timing, method of intervention delivery, and the presence of a control group).

Quality assessment was performed in duplicate independently by ID, FZ, and MS. Rating discrepancies were resolved by a third study team member (SCC). Quality assessment was conducted using the JBI’s critical appraisal tools for case–control studies, cohort studies, and cross-sectional studies. (Moola et al., 2020) Each JBI checklist item was answered with a “yes,” “no,” “unclear,” or “not applicable” response. Total scores were determined by summing “yes” responses. Study quality was assessed using the National Institutes of Health Study Quality Assessment Tools for the single-arm intervention study. (National Institutes of Health, 2020) Responses to each criterion were categorized as “yes,” “no,” “not reported,” “cannot determine,” or “not applicable” and the single-arm intervention was then assigned a risk of bias rating as low (75–100%), moderate (25–75%), or high (0–25%). For all appraisal tools, total scores were calculated based on responses to applicable questions only.

Meta-analyses were conducted when possible. Effect sizes (Cohen’s *d*) were meta-analysed using R statistical software and the “metafor” package when data from three or more studies were available. (Viechtbauer, 2010; R Core Team, 2022) Cohen’s *d* effects were classified as small (|*d*| = 0.20–0.35), moderate (|*d*| = 0.35–0.65), or large (|*d*| ≥ 0.65). (Cohen, 1988) Effect size estimates were based on a random-effects model. Hartung–Knapp adjustments were used to calculate effect sizes and confidence intervals due to the relatively small number of studies and the wide variation in instruments used to measure mental health symptoms in included studies. (Hartung & Knapp, 2003) Variations in effect sizes were assessed using both the *Q* and *I*^2^ statistics. The *Q* statistic tests the assumption of a single effect size, while *I*^2^ quantifies the proportion of true variability (heterogeneity) between studies. Standard cut-offs (low: 0–25%, moderate: 26–50%, and high: 51–75% or more) were used to interpret heterogeneity levels. (Thompson & Higgins, 2002; Higgins et al., 2003) For studies in which data were insufficient for meta-analyses, study results were synthesized narratively.

Microbial diversity was assessed using two key metrics: alpha and beta diversity. Alpha diversity quantifies the richness and evenness of species within a single bacterial sample, reflecting the local diversity of the bacterial community. Common alpha diversity metrics included species richness (e.g., observed species number) and diversity indices (e.g., Shannon index and Simpson index) that incorporate richness and abundance information. Beta diversity assesses the compositional dissimilarity between bacterial communities in different samples. It addresses how bacterial communities differ in species composition and abundance across environments. Beta diversity metrics can be based on the presence/absence data (e.g., Jaccard index) or the abundance data (e.g., Bray–Curtis dissimilarity).

The taxonomic synthesis identified operational taxonomic units (OTUs) that consistently exhibited an association or demonstrated efficacy across multiple studies for internalizing problems, depression, and anxiety. By focusing on OTUs that exhibited consistent patterns across studies, we aimed to identify associations between gut microbiota and depression, anxiety, and internalizing symptoms. Phylogenetic trees were created using the “ape” package in R. (Paradis et al., 2019)

Consistent with the integrated knowledge translation plan, and to inform the knowledge translation framework and designed to support a youth-informed dissemination plan, youth research partners contributed unique perspectives on the study findings, their implications, and relevance to lived youth experiences. Five youth research partners (aged 16–21 years, 60% female) were self-nominated based on interest and availability. Youth research partners with and without lived experience of depression/anxiety were recruited from an existing pool of youth research advisors at SickKids Hospital, Toronto, Canada, and from the community. Youth reviewed preparatory materials on the gut microbiome and mental health to ensure informed participation. Youth partners made recommendations for dissemination and future research based on their understanding of how the research could best reach and benefit young people.

## Results

Eight studies representing 2,865 participants (mean age 11.4 years [SD = 4.3]; 43% female) met the criteria for inclusion (Figure 1). All studies were published between 2021 and 2024. Seven were observational in design – five cross-sectional and two longitudinal cohort studies – and one was a single-arm 8-week probiotic intervention. The studies investigated associations between the gut microbiome and internalizing problems (*n* = 3), depression (*n* = 4), and anxiety (*n* = 2), with some studies addressing multiple disorders. Two studies were conducted among children under 5 years of age, three among children between 5 and 10 years of age, and three among adolescents between 10 and 15 years of age. Case–control studies comparing clinically diagnosed participants to healthy controls were conducted in China (*n* = 3). The cross-sectional studies were conducted in the Netherlands (*n* = 2), Canada (*n* = 1), and the United States (*n* = 1) among community samples with sub-threshold scores for internalizing problems, anxiety, and depression. The sole intervention study was conducted in China. Sample sizes ranged considerably (*n* = 21–1,784). All eight included studies analysed faecal samples using 16S ribosomal RNA gene sequencing to characterize bacterial and archaeal communities; none reported amplicon sequence variants (ASVs) or metagenomic data. Detailed study characteristics are presented in Table 1.

**Table 1.** Summary of study characteristics

References	Disorder	Country	Sample size	% Female	Mean age, SD (years)	Age range (years)	Diagnosis (by clinician)	Mental health symptom measure	Mean score (clinical cut-off)	Informant	Quality assessment score
Matched cross-sectional (*n* = 4)											
Cai et al. (2022)	Depression	China	48	Case: 75% Control: 66.7%	Case: 15.0; Control: 15.0	12–18	Depression diagnosis with DSM–5	HAMD	NR	Child	8/9
Ling et al. (2022)	Depression	China	140	54%	Case: 8.84 (1.89); Control: 9.27 (2.11)	6–12	Depression diagnosis with DSM–5	HAMD	Case: 24.0 Control: 4.2 (HAMD >7)	Child	8/9
Zhou et al. (2022)	Depression	China	171	Case: 62.2%; Control: 53.4%	Case:13.7 (2.6); Control: 13.5 (3.1)	12–18	Depression diagnosis with ICD–10; Controls screened using the MINI	SDS SAS	SDS: Case: 76.3; Control: 33.7 (SDS ≥ 50) SAS: Case: 63.0; Control: 31.1 (SAS ≥ 36)	Child	8/9
Cross-sectional, (*n* = 2)											
Kraaij et al. (2023)	Internalizing	Netherlands	1784	48.80%	9.8 (0.29)	6–18	No	CBCL	4.79 (T-score ≥ 65)	Parent	8/8
van de Wouw et al. (2022)	Internalizing	Canada	248	47.60%	4.37	3–5	No	CBCL	50 (T-score ≥ 65)	Parent	8/8
Longitudinal cohort (*n* = 2)											
Laue et al. (2022)	Internalizing	USA	260	44.60%	3.2 ± 0.3	0–3	No	BASC–2	47.6 (BASC–2 ≥ 70)	Parent	7/9
	Anxiety							BASC–2	50.4 (BASC–2 ≥ 70)		
	Depression							BASC–2	48 (BASC–2 ≥ 70)		
Ou et al. (2024)	Internalizing	Netherlands	193	NR	14.46 (0.17)	14	No	SDQ	4.14 (SDQ > 7)	Child	9/9

Reference	Disorder	Country	Sample size	% Female	Mean age, SD (years)	Age range (years)	Diagnosis (by physician)	Mental health symptom measure	Mean score (clinical cut-off)	Informant	Intervention	Risk of bias rating
Intervention (*n* = 1)												
Dong et al. (2020)	Anxiety	China	21	0%	9.1 ± 1.80	NR	No	MRF	Week 0. MRF: Cognition anxiety 7.62; Somatic anxiety 6.76 Week 8. MRF: Cognition anxiety 4.30; Somatic anxiety 6.15 (MRF: higher score = increased anxiety)	Child	8 weeks with a probiotic (*Bifidobacterium animalis*)	Moderate

Abbreviations: BASC: Behaviour Assessment System for Children; CBCL: Child Behavior Checklist; DSM-5: Diagnostic and Statistical Manual of Mental Disorders-Fifth Edition; HAMD: Hamilton Depression Scale; ICD-10: International Statistical Classification of Diseases and Related Health Problems-10; MINI: Mini-International Neuropsychiatric Interview; MRF: Mental Readiness Form; SAS, Self-Rating Anxiety Scale; SAS-A: Social Anxiety Scale for Adolescents; SDQ: Strengths and Difficulties Questionnaire; SDS: Self-rating Depression Scale; NR: none reported.

## Gut microbiome evidence synthesis

### Alpha and beta diversity

Diversity findings are summarized in Table 2. Seven observational studies reported measures of alpha diversity, of which the Shannon index was the most frequently reported. Other reported alpha diversity indices included Simpson (*n* = 5), Chao1 (*n* = 4), ACE (*n* = 4), and Species Observed (*n* = 3). The sole intervention study reported all five alpha diversity indices. Six studies assessed the variation in gut microbiome composition (beta diversity) abundance data using various metrics. Weighted UniFrac distance (*n* = 3) and unweighted UniFrac distance (*n* = 3) were the most frequent metrics. Other metrics included pairwise Euclidean distance matrix (*n* = 1) and Bray–Curtis distances (*n* = 1). Only one study reported beta diversity based on the presence/absence data using the Jaccard Index. Given the limited number of studies addressing each disorder, heterogeneity in participant age ranges, and variation in reported beta diversity metrics, a meta-analysis of beta diversity could not be conducted.

**Table 2.** Summary of alpha and beta diversity findings

Diversity	Disorder	Number of studies (study design)	Findings	Meta-analyses/overall findings
Alpha	Depression	*n* = 3 studies (case–control) (Cai et al., 2022; Ling et al., 2022; Zhou et al., 2022)	Case–control studies: Effect sizes of alpha diversity (ACE, Chao 1) were medium to large for older adolescents and large for younger children in opposing directions. Effect sizes of alpha diversity (Shannon Index) were medium to large for older adolescents and small for younger children in opposing directions	No significant difference in alpha diversity indices between participants diagnosed with depression and healthy controls Pooled effect sizes (Cohen’s *d*) ranged from −0.04 to 0.31, with all confidence intervals including zero (Figure 2A–D) for ACE, Chao 1, and the Shannon Index
Alpha	Anxiety	*n* = 1 (longitudinal observational) (Laue et al., 2022); *n* = 1 (interventional study) (Dong et al., 2020)	Longitudinal study: No association was observed between alpha diversity at 6 weeks and anxiety at 3 years (Shannon: *B* = −0.63, 95% CI [−1.97, 0.71]; inverse Simpson: *B* = −0.96, 95% CI [−2.28, 0.35]) or between alpha diversity at 2 years and anxiety at 3 years (Shannon: *B* = 1.03, 95% CI [−0.60, 2.66]; inverse Simpson: *B* = 1.13, 95% CI [−0.45, 2.70]) Intervention study: No significant difference (*p* = 0.73 to *p* = 0.09) in any of the alpha diversity indices before and after the intervention	No significant association between alpha diversity and anxiety
Alpha	Internalizing problems	*n* = 2 (cross-sectional) (Laue et al., 2022; van de Wouw et al., 2022); *n* = 1 (longitudinal) (Laue et al., 2022)	Cross-sectional studies: Combined effect size (z) of the Shannon Index *z* = −0.07, 95% CI [−0.49, 0.38]), *Q* = 1.55, *I*^2^ = 35.38% Longitudinal cohort: While the entire sample showed a negative association between alpha diversity at6 weeks and internalizing problems at 3 years using the Simpson index: *B* = −1.19, 95% CI [−2.36, −0.02] but not the Shannon Index: *B* = −0.83, 95% CI [−2.02, 0.37], the association was not present at later time points. Boys with lower alpha diversity at 6 weeks exhibited a significantly stronger negative association with internalizing problems at 3 years compared to the entire sample (Shannon: *B* = −2.19, 95% CI [−3.71, −0.66]; Simpson: *B* = −2.57, 95% CI [−4.08, −1.07])	No overall significant association between alpha diversity and internalizing problems For boys, lower alpha diversity at 6 weeks was significantly associated with internalizing symptoms at 3 years of age
Beta	Depression	*n* = 2 (case–control) (Ling et al., 2022; Zhou et al., 2022) *n* = 1 (cross-sectional) (Kraaij et al., 2023) *n* = 1 (longitudinal) (Laue et al., 2022)	Case–control studies: Significant decrease in bacterial beta diversity in the depression group compared to healthy controls Cross-sectional study: No significant association. Longitudinal study: No significant associations between bacterial beta diversity at 6 weeks, 1 year, or 2 years, and depression scores at 3 years	Mixed results depending on the study design
Beta	Anxiety	*n* = 1 (longitudinal) (Laue et al., 2022) *n* = 1 (interventional) (Dong et al., 2020)	Longitudinal study: No significant associations between bacterial beta diversity at various time points and anxiety scores at a later age. For boys, beta diversity at 6 weeks was associated with anxiety scores at 3 years in the fully adjusted model (*R*^2^ = 0.012, *p* = 0.02) but not at later time points Intervention study: No significant difference in beta diversity before and after the intervention	No overall significant association between beta diversity and anxiety Among boys, beta diversity at 6 weeks was associated with anxiety scores at 3 years
Beta	Internalizing problems	*n* = 1 (cross-sectional) (Kraaij et al., 2023); *n* = 2 (longitudinal) (Laue et al., 2022; Ou et al., 2024)	Cross-sectional study: No significant association between beta diversity and internalizing problems Longitudinal studies: No significant association between beta diversity at 6 weeks, 1 year, or 2 years, and internalizing problem scores at 3 years	No significant association between beta diversity and internalizing problems

**Figure 2.**
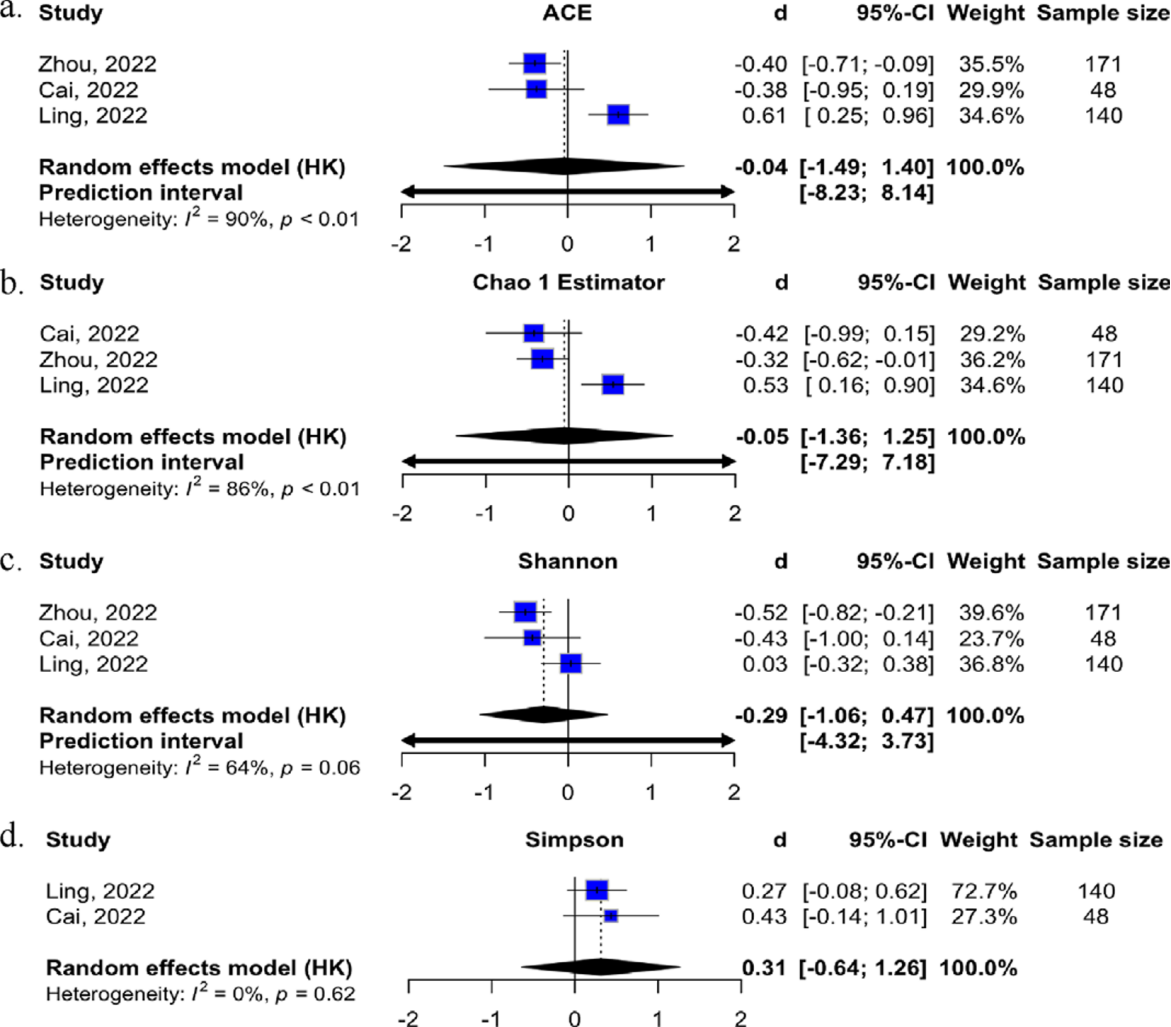
Meta-analyses of the alpha diversity indices for depression. (A) ACE, (B) Chao 1, (C) Shannon, and (D) Simpson in children and adolescents.

Alpha diversity indices were not significantly associated with depression (Figure 2). While two studies, including older adolescents (aged 12–18 years), reported a negative association between alpha diversity indices (ACE, Chao 1, and Shannon) and depression, the study with younger children (aged 6–12 years) found a positive association. No significant association between alpha diversity and either anxiety or internalizing symptoms was found among the included studies. Studies of the association between beta diversity and depression were mixed, with two studies observing a positive association (Ling et al., 2022; Zhou et al., 2022) and two studies observing a negative association (Laue et al., 2022; Kraaij et al., 2023), in children and adolescents. None of the included studies observed an association between beta diversity and anxiety or internalizing problems. Among boys, alpha and beta diversity in infancy (6 weeks of age) was associated with anxiety at 3 years (Laue et al., 2022).

## Taxonomic synthesis

**Figure 3.**
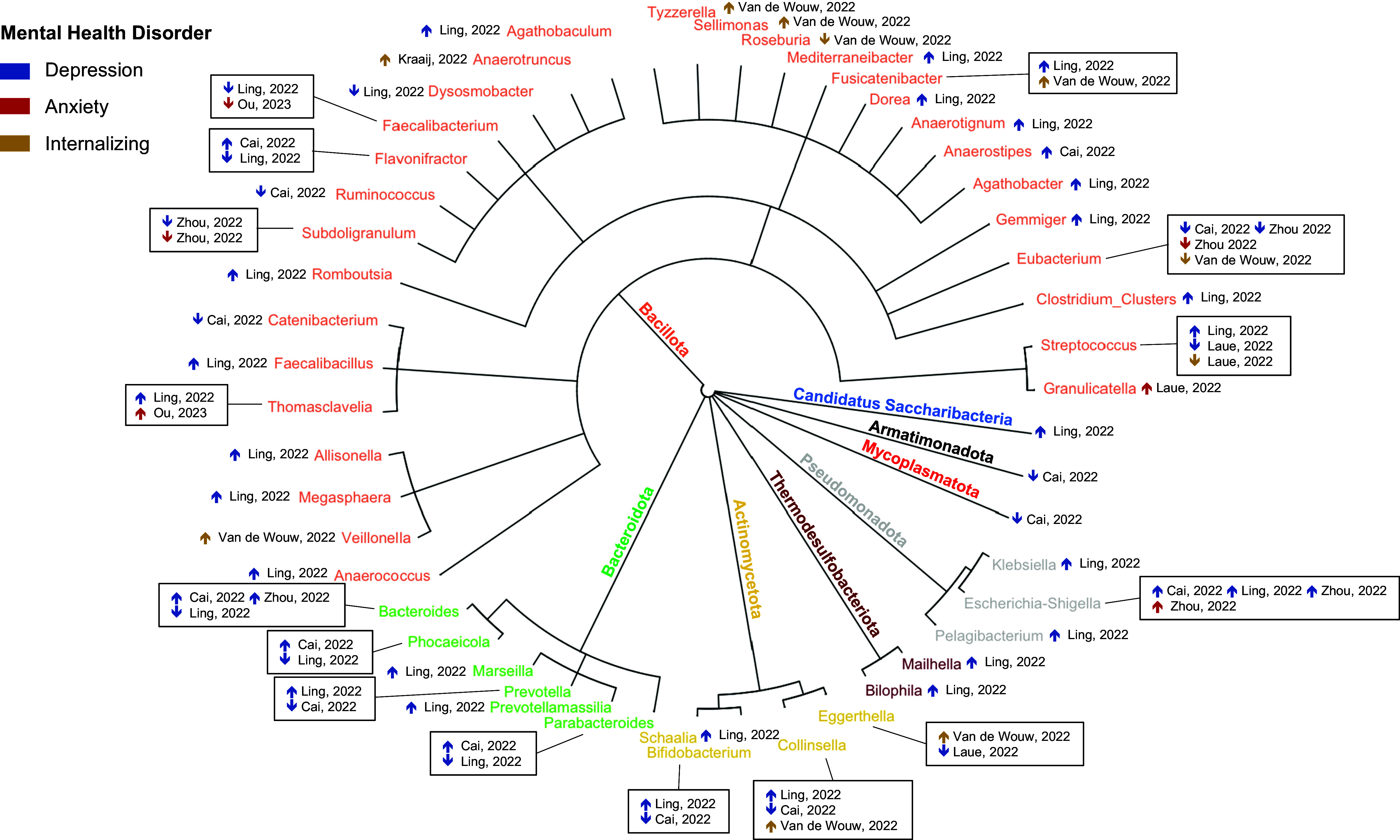
Reported abundance of gut operational taxonomic units in children and adolescents (*n* = 8). The outer circle represents the genus level, with colours distinguishing the phylum. Dark blue arrows indicate studies of depression, red arrows indicate studies of anxiety, and gold arrows indicate studies of internalizing problems. Each box in the figure shows the results from multiple studies per species. ↑ indicates higher abundance in children and adolescents with the disorder, while ↓ indicates lower abundance among those with the disorder.

Among the 7 observational studies, 45 bacterial taxa were reported at the genus level, 12 at the family level, 2 at the class level, and 1 at the phylum level **(** Figure 3). Fifteen taxa were reported in more than one study. Specific genera showed consistent associations with internalizing symptoms, depression, and anxiety. Two studies reported increased abundance of genera within the class Clostridia (phylum Bacillota), including *Fusicatenibacter* and *Thomasclavelia.* Similarly, increased abundance of the *Escherichia-Shigella* group (order Enterobacterales, phylum Pseudomonadota) was observed in depression. Within Clostridia, decreased abundance was reported for *Subdoligranulum* and *Faecalibacterium* (order Oscillospirales), and Eubacterium (order Eubacteriales).

Inconsistencies were noted across genera from multiple phyla. For example, *Bacteroides* (phylum Bacteroidota) was reported as increased in two studies and decreased in one for depression. *Collinsella* (phylum Actinomycetota) and *Streptococcus* (phylum Bacillota) were each reported as both increased and decreased across studies. Other taxa were reported inconsistently or appeared in only a single study, limiting definitive conclusions.

The 8-week intervention study administering *Bifidobacterium animalis* reported no significant changes in alpha or beta diversity but did find increased abundance of the family *Bifidobacteriaceae.*

## Quality assessment

Despite high-quality scores, all three case–control studies (Cai et al., 2022; Ling et al., 2022; Zhou et al., 2022) were downgraded because the psychometric assessment tools used lacked construct validity for the younger age group. Both of the prospective cohort study scores (Laue et al., 2022; Zhou et al., 2022; Ou et al., 2024) were downgraded as neither reported strategies for addressing loss to follow-up. Two cross-sectional studies (van de Wouw et al., 2022; Kraaij et al., 2023) also received high-quality assessment scores and were not downgraded for any criteria. The single-arm intervention study (*n* = 1) had a moderate risk of bias due to a small sample size and limited generalizability, as it focused solely on young male athletes. No studies were eliminated from the evidence synthesis based on methodological quality since this was not one of the *a priori* exclusion criteria (Supplementary Tables 2–5).

## Youth engagement

The youth engagement session was conducted to explore young people’s understanding of the gut microbiome and its potential association with mental health. While youth demonstrated familiarity with general healthy eating guidelines, specific knowledge regarding the gut microbiome was limited. Their sources for nutrition information were diverse, encompassing parents, coaches, and social media platforms. They were eager to learn about the impact of diet and the microbiome on mental health. Additionally, participants expressed a desire for the practical application of research findings that could directly benefit their mental well-being. Youth research partners indicated that while the connection between gut health and health was new information for them, the concept was interesting and important to them, and made intuitive sense once explained. This feedback confirmed that this research addresses a knowledge gap relevant to young people and validated the importance of translating gut microbiome research into accessible formats for youth audiences. In addition, the youth partners provided insights into the use of specific language and terminology to optimize the effectiveness of communication with youth audiences. Moreover, our youth research partners confirmed that our focus on practical applications aligned with youth priorities for mental health research. The engagement session further aimed to identify knowledge dissemination avenues. Integrating information into existing resources such as mental health programmes and school curricula was identified as a favoured approach. Doctors’ offices and schools were viewed as valuable channels for reaching parents. Recipe books containing meal plans that promote mental health were also viewed positively. However, cost and convenience were recognized as potential barriers to the adoption of healthy eating habits. Interestingly, some participants expressed openness to contributing stool samples for research purposes, but anonymity and a discreet return process were of the utmost importance, with suggestions including the use of opaque, pre-labelled packaging.

## Discussion

This systematic review investigated the connection between gut microbiome and mental health in children and adolescents among eight included studies. Depression was the most frequently explored mental health outcome, followed by internalizing problems and anxiety. While most studies relied on community samples, three included studies employed clinical diagnoses for depression. Heterogeneity in factors such as sample size and age range likely reflects the early stage of this field research and underscores the necessity for future investigations employing larger, more diverse cohorts. The relatively small number of studies, particularly within each age group, precluded stratified analyses (e.g., comparing younger children and adolescents or comparing Asian to non-Asian populations). Geographically, studies were relatively balanced, with 50% conducted in East Asia and the remainder in Europe and North America. However, for specific outcomes, such as alpha diversity, all contributing studies were conducted in China, restricting our ability to explore regional variability. As factors such as diet, climate, and cultural practices may influence microbiome composition, future research with more geographically distributed data across analyses is warranted to better assess potential region-specific associations. All were published after 2021, reflecting the growing interest in the gut microbiome’s role in mental health and the infancy of the field.

Although preliminary, this review offers insights into potential relationships between specific bacterial genera and gut microbiome diversity (measured by alpha and beta diversity) in relation to internalizing problems, depression, and anxiety in youth. No significant associations were observed between alpha diversity and any of the mental health outcomes overall. However, possible age-dependent effects were noted: studies of older adolescents (12–18 years) reported lower alpha diversity in individuals with depression (Cai et al., 2022; Zhou et al., 2022), while one study of younger children (6–12 years) found higher alpha diversity associated with depression. (Ling et al., 2022) These findings suggest potential age-related dynamics in the gut microbiome–mental health relationship. The influence of beta diversity on depression, anxiety, and internalizing problems remains unclear, with mixed results. For example, one study found that lower beta diversity at 6 weeks of age was associated with higher anxiety at age three in boys, (Laue et al., 2022) while others reported no significant associations. Additionally, substantial heterogeneity in the diversity indices and metrics used – particularly for beta diversity – limited comparability across studies.

Several bacterial genera exhibited consistent signals of association with depression, anxiety, and internalizing problems. The most consistent evidence was observed for the phylum Pseudomonadota, particularly the *Escherichia-Shigella* group, which was found in higher abundance in individuals with depression. These well-known pathogens are associated with high-glucose, high-protein, and high-fast-food diets, and their presence in higher numbers is potentially indicative of gut dysbiosis. (Cláudia-Ferreira et al., 2023; Rodrigues et al., 2023) Similarly, increased abundance of genera within the phylum Bacillota, such as *Fusicatenibacter* and *Thomasclavelia*, was observed in studies reporting internalizing problems and depression. *Fusicatenibacter* are anaerobic sugar fermenters linked to unhealthy eating behaviours, obesity, and processed food consumption. (Takada et al., 2013; Matsuyama et al., 2019; Medawar et al., 2021) *Thomasclavelia* has been shown to enhance sugar and fat absorption in mice on high-fat diets, suggesting obesogenic potential. (Woting et al., 2014) Conversely, decreased abundance of *Subdoligranulum*, *Eubacterium*, and *Faecalibacterium* – also within Bacillota – was consistently reported with depression, anxiety, and internalizing symptoms. These genera are considered beneficial due to their roles in carbohydrate fermentation and SCFA production. *Subdoligranulum* is more abundant in individuals with higher carbohydrate intake (Holmstrøm et al., 2004; Cani et al., 2021), while low levels of *Eubacterium* are associated with fibre-poor diets and inflammation. (Mukherjee et al., 2020) *Faecalibacterium* is similarly linked to diets rich in non-digestible carbohydrates and is known for its anti-inflammatory properties. (Cani et al., 2021; Maioli et al., 2021) Both, *Faecalibacterium* and *Subdoligranulum* are known to produce beneficial SCFAs that promote gut health and are anti-inflammatory. (Verhoog et al., 2019) In contrast, findings for genera within the phyla Bacteroidota and Actinomycetota were inconsistent. For example, *Bacteroides* was reported as increased in two studies and decreased in one for depression. Similarly, *Collinsella* showed both increased and decreased abundance across studies. Even within Bacillota, some genera, such as *Streptococcus*, exhibited conflicting results. These inconsistencies may stem from natural variability in gut microbiome composition across populations, methodological differences, or cohort-specific factors.

It is important to acknowledge the broader methodological limitations of 16S ribosomal RNA (rRNA) sequencing, which was the sole approach used across the included studies. While 16S rRNA sequencing is useful for profiling microbial communities, it typically lacks the resolution to identify microbes to strain or even species level and provides limited functional insights into microbial activity. Additionally, the use of OTUs rather than more precise ASVs may obscure taxonomic resolution, as OTUs can group functionally diverse organisms. This limitation reduces the ability to detect subtle but potentially meaningful microbial differences. These methodological constraints likely contribute to inconsistencies across studies and limit the interpretation of compositional findings related to mental health outcomes. As such, our conclusions should be interpreted with caution and viewed as preliminary. Despite these limitations, the findings collectively suggest a potential association between increased depressive symptoms and a gut microbiome characterized by reduced abundance of beneficial SCFA-producing bacteria and increased abundance of taxa associated with inflammation and poor dietary patterns.

This systematic review has some methodological strengths. Notably, it employed a comprehensive approach, encompassing internalizing problems, depression, and anxiety, which are among the most prevalent among children and adolescents. Furthermore, the search and review process adhered to rigorous methods. The broad search strategy ensured comprehensive coverage of the literature by capturing a large number of records without imposing unnecessary restrictions on publication date or geographical origin. Despite its methodological strengths, some limitations warrant consideration when interpreting the findings. A high degree of heterogeneity among the included studies posed a challenge. This heterogeneity prevented the use of meta-analysis, specifically for alpha diversity and anxiety or internalizing problems, as well as beta diversity and mental health disorders investigated. Additionally, the limited number of studies precluded the use of meta-regression or subgroup analysis. These limitations restricted the ability to draw definitive conclusions about the overall strength of the evidence. As the nascent field of gut microbiome and internalizing problems, such as depression and anxiety, grows and yields more homogenous studies, more robust statistical analyses will become possible.

To propel research on the gut microbiome and its connection to internalizing problems like depression and anxiety, future studies should prioritize several key areas. First, larger studies with clinically confirmed diagnoses across the spectrum of internalizing problems are crucial. Second, conducting studies with more specific age groups might reveal the details necessary to determine potential variations in the gut microbiome–mental health association across developmental periods. This would be crucial in determining if the link manifests differently in younger children compared to adolescents. Third, analysis of 16S rRNA survey data should focus on the use of ASVs, which provide an improved definition of taxa relative to OTUs, to better allow cross-comparisons across studies. Fourth, studies need to consider the inclusion of technologies, such as whole microbiome shotgun DNA and RNA sequencing (metagenomics and meta-transcriptomics) and metabolomics, on at least a subset of samples to deliver functional insights through defining the presence, abundance, and expression of biochemical pathways encoded by the communities. Indeed, metabolites, being less susceptible to environmental influences than microbial taxa, identified through metabolomics, may serve as more robust biomarkers. A parallel focus on inflammatory markers, both as mechanistic drivers and potential biomarkers, can deepen our understanding of microbiome–host interactions. Together, these technologies will enable a comprehensive characterization of the microbiome and its role in health and disease. Finally, longitudinal studies with larger, geographically and culturally diverse populations are essential. Moreover, by tracking microbiome changes over time and across cultures, such studies can illuminate causal relationships and identify potential regional variations within the gut microbiome–mental health axis. This comprehensive approach will significantly enhance our understanding of this intricate connection.

While methodological rigour is essential, meaningful progress in this field also hinges on inclusive and participatory research practices. Consistent with our integrated knowledge translation approach, the youth engagement component of this study highlighted the importance of involving young people in research endeavours and provided valuable insights that enhanced the contextual relevance of our study. Youth research partners validated the importance of our research questions to their lived experiences and confirmed that our findings align with their perspectives on the connections between gut health and mental health. They provided specific insights into terminology and concepts that resonate with young people, helping to ensure our research remains accessible and meaningful to this population. The willingness expressed by youth research partners to provide stool samples suggests a growing acceptance of this practice among youth, which is particularly noteworthy given that sample collection has historically been a barrier to microbiome research participation. The perspectives of our youth research partners also align with a recent study of 90 adolescents (aged 13–19 years), in which 89% contributed stool samples. (Leung et al., 2024) Thus, the insights gained from our youth research partners provide valuable information for developing practical intervention strategies that translate gut microbiome research into tangible tools capable of improving youth mental health outcomes.

## Conclusions

This systematic review identified consistent patterns of abundance across studies in bacterial taxa, which are preliminarily suggestive of a gut microbiome in dysbiosis in children and adolescents with depression and anxiety. Possible age-related patterns were found for alpha diversity and depression. Future research with larger, geographically diverse samples, clinically confirmed diagnoses, and a focus on microbial metabolism and gut metabolites is crucial to strengthen the evidence in this evolving field. Youth research partners not only confirmed the personal relevance of our research and validated our findings about gut–mental health connections, but also expressed strong interest in dietary implications, demonstrated readiness to participate in future microbiome research studies through stool sample provision, and identified trusted adults (parents, doctors, teachers, and coaches) as key channels for effective dissemination to youth audiences.

## Supporting information

Campisi et al. supplementary material Campisi et al. supplementary material

## Data Availability

Data described in the manuscript, code book, and analytic code will be made available upon request to Daphne J. Korczak, 1145 Burton Wing, Department of Psychiatry, Hospital for Sick Children, 555 University Avenue, Toronto, Ontario, Canada M5G 1X8; Tel 416 813–6936; Fax 416–813-5236; email daphne.korczak@sickkids.ca

## Abbreviations

ASVs: amplicon sequence variants
JBI: Joanna Briggs Institute
OTUs: operational taxonomic units
PRISMA: Preferred Reporting Items for Systematic Reviews and Meta-Analyses

## Supplementary material

The supplementary material for this article can be found at http://doi.org/10.1017/gmb.2025.10013.

## Registry number for systematic reviews

PROSPERO CRD 42023476178.
