# Supplementary material for: The gut microbiome and child and adolescent depression and anxiety: a systematic review and meta-analysis with youth consultation
Source: Gut Microbiome (Camb). 2025 Sep 1;6:e17. doi: 10.1017/gmb.2025.10013 (PMC12509154; doi:10.1017/gmb.2025.10013)
Supplement: Campisi et al. supplementary material [file S2632289725100133sup001.docx]

**Supplementary Table 1**. SEARCH SUMMARY

| Database & Interface | Date Searched | Results |
| --- | --- | --- |
| Ovid MEDLINE: Epub Ahead of Print, In-Process & Other Non-Indexed Citations, Ovid MEDLINE® Daily and Ovid MEDLINE® <1946-Present> | Nov. 8 2023 | 3215 |
| Embase Classic+Embase <1947 to 2023 November 07> | Nov. 8 2023 | 5070 |
| Interface - EBSCOhost Research Databases Search Screen - Advanced Search Database - CINAHL Plus with Full Text | Nov. 9 2023 | 535 |
| Interface - EBSCOhost Research Databases Search Screen - Advanced Search Database - Child Development & Adolescent | Nov. 6 2023 | 30 |
| APA PsycInfo | Nov. 6 2023 | 394 |
| Elsevier Scopus | Nov. 6 2023 | 4001 |
| Wiley Cochrane Library | Nov. 7 2023 | 2012 |
| **Final Results (prior to deduplication)** | | 15257 |

SEARCH STRATEGIES

**Database: Ovid MEDLINE: Epub Ahead of Print, In-Process & Other Non-Indexed Citations, Ovid MEDLINE® Daily and Ovid MEDLINE® <1946-Present>**

**Search Strategy:**
**1** Depression/ or depressive disorder/ or depression, postpartum/ or depressive disorder, major/ or depressive disorder, treatment-resistant/ or dysthymic disorder/ or premenstrual dysphoric disorder/ or seasonal affective disorder/ or Mood Disorders/ or Mental Health/ or Mental Disorders/ (483781)
2 (depress* or dysthymia or sad or sadness or saddest or sadder or melanchol* or miser* or unhappiness or unhappy or emotion* or mood* or dysthymi* or MDD or affective* or dysphoric* or mental* or psychiat* or psychopath* or behavio?r* or anxiodepress* or sorrow*).tw,kf. (2782236)
3 internal*.tw,kf. (602236)
4 anxiety disorders/ or anxiety, separation/ or anxiety/ or dental anxiety/ or performance anxiety/ or test anxiety/ (145338)
5 (anxiet* or anxious* or hypervigilan* or nervous* or angst* or worry or worries or apprehensi* or uneasiness or unease or fear or fears or distress* or phobia*).tw,kf. (886359)
6 or/1-5 [depression, anxiety, internalization] (3919308)
7 Gastrointestinal Microbiome/ or Fecal Microbiota Transplantation/ or Brain-Gut Axis/ (41461)
8 firmicutes/ or acidaminococcus/ or Bacteroidetes/ or Lactobacillus/ (22569)
9 microbiota/ and (gastrointestinal tract/ or intestines/ or intestine, large/ or anal canal/ or cecum/ or colon/ or colon, ascending/ or colon, descending/ or colon, sigmoid/ or colon, transverse/ or rectum/ or intestine, small/ or duodenum/ or "ampulla of vater"/ or "sphincter of oddi"/ or ileum/ or jejunum/ or lower gastrointestinal tract/ or stomach/) (4033)
10 (firmicute* or acidaminococc* or Bacteroidete* or Lactobacill* or lactobacteri* or lactococc* or bifidobacter* or streptococcus thermophilus or bacillus subtilis).tw,kf. (115238)
11 ((gastro* or gut* or gastric* or intestin* or stomach* or fecal* or faecal or f?ece* or stool or colon* or bowel* or ileum or ileal or duoden* or jejun* or digestive or cecum or sigmoid or splenic flexure or rectum or anal canal or ampulla of vater or ileocecal valve or sphincter or cardia or forestomach or pylorus) adj6 (microb* or flora or microflor* or micro-flor* or microorgan* or micro-organ* or microecolog* or micro-ecolog* or dysbios* or dysbacterios* or disbacteri* or disbios* or symbio* or Actinobacteri* or Proteobacteri* or Escherichi* or Candida or Saccharomyces or Aspergillus or Penicillium or Rhodotorula or Trametes or Pleospora or Sclerotinia or Bullera or Galactomyces)).tw,kf. (126660)
12 ((f?ecal or stool or f?ece* or microb*) adj4 (transplant* or therap* or transfer* or infusion*)).tw,kf. (15393)
13 (gut adj2 brain).tw,kf. (9077)
14 enteric bacteria*.tw,kf. (4139)
15 Prebiotics/ or Probiotics/ (27023)
16 (probiotic* or prebiotic* or psychobiotic*).tw,kf. (48858)
17 or/7-16 [gut microbiome/ probiotics] (259032)
18 pediatrics/ (57964)
19 young adult/ (1016709)
20 adolescent/ (2225775)
21 child/ (1931193)
22 child, preschool/ or schools/ or universities/ or students/ or minors/ (1139552)
23 infant/ or infant, newborn/ or infant, large for gestational age/ or infant, low birth weight/ or infant, small for gestational age/ or infant, very low birth weight/ or infant, extremely low birth weight/ or infant, postmature/ or infant, premature/ or infant, extremely premature/ (1259481)
24 (infant* or baby or babies or newborn* or neonat* or minor or minors or toddler* or boy or boys or boyhood or girl or girls or girlhood or kid or kids or kiddie or child* or schoolchild* or adolescen* or preadolescen* or juvenil* or youth* or teen* or preteen* or underage* or under age* or pubescen* or prepubescen* or pre mature* or premature* or puberty or p?ediatric* or school* or schoolage*).tw,kf. (3417542)
25 (young* adj3 (adult* or person* or individual* or people* or population* or man or men or wom?n or male* or female*)).tw,kf. (339101)
26 (youngster* or preschool* or kindergar?en* or freshm?n* or junior* or soph?more* or senior* or highschool* or colleg* or universit* or student* or undergrad* or middleschool*).tw,kf. (976315)
27 ((grade* or form*) adj3 (one or first or two or second or three or third or four* or five or fifth or six* or seven* or eight* or nine* or ninth or ten* or eleven* or twelfth or twelve or "1" or "2" or "3" or "4" or "5" or "6" or "7" or "8" or "9" or "10" or "11" or "12")).tw,kf. (608439)
28 all age*.tw,kf. (50983)
29 or/18-28 [children] (6966711)
30 6 and 17 and 29 (3215)

**Database: Embase Classic+Embase <1947 to 2023 November 07>**
**Search Strategy:**
**1** depression/ or dysthymia/ (505585)
2 adolescent depression/ or agitated depression/ or atypical depression/ or chronic depression/ or depressive psychosis/ or endogenous depression/ or involutional depression/ or major depression/ or masked depression/ or melancholia/ or minor depression/ or "mixed anxiety and depression"/ or organic depression/ or post-stroke depression/ or postoperative depression/ or premenstrual dysphoric disorder/ or reactive depression/ or recurrent brief depression/ or seasonal affective disorder/ or subsyndromal depression/ or treatment resistant depression/ or postnatal depression/ (107898)
3 (depress* or dysthymia or sad or sadness or saddest or sadder or melanchol* or miser* or unhappiness or unhappy or emotion* or mood* or dysthymi* or MDD or affective* or dysphoric* or mental* or psychiat* or psychopath* or behavio?r* or anxiodepress* or sorrow*).tw,kf. (3489073)
4 internal*.tw,kf. (775373)
5 anxiety disorder/ or acute stress disorder/ or anxiety neurosis/ or distress syndrome/ or generalized anxiety disorder/ or "mixed anxiety and depression"/ or panic/ or phobia/ or separation anxiety/ or internalizing disorder/ (213210)
6 dental anxiety/ or dental phobia/ or test anxiety/ or performance anxiety/ (3922)
7 (anxiet* or anxious* or hypervigilan* or nervous* or angst* or worry or worries or apprehensi* or uneasiness or unease or fear or fears or distress* or phobia*).tw,kf. (1230550)
8 or/1-7 (5014187)
9 intestine flora/ or colon flora/ (103592)
10 fecal microbiota transplantation/ (9594)
11 brain-gut axis/ (2388)
12 firmicutes/ or bacteroidetes/ or Lactobacillus/ (58900)
13 microflora/ and (intestine/ or gastrointestinal tract/ or lower gastrointestinal tract/ or upper gastrointestinal tract/ or large intestine/ or cecum/ or colon/ or sigmoid/ or transverse colon/ or colon tenia/ or descending colon/ or splenic flexure/ or ascending colon/ or rectum/ or rectum epithelium/ or rectum gland/ or anal canal/ or small intestine/ or ampulla of Vater/ or ileocecal valve/ or ileum/ or ileum epithelium/ or terminal ileum/ or upper ileum/ or jejunum/ or jejunum epithelium/ or Oddi sphincter/ or small intestine epithelium/ or small intestine muscle/ or duodenal muscle/ or ileal muscle/ or jejunal muscle/ or small intestine wall/ or duodenum/ or duodenum epithelium/ or lower gastrointestinal tract/ or cardia/ or forestomach/ or gastroesophageal junction/ or greater curvature of the stomach/ or lesser curvature of the stomach/ or pyloric sphincter/ or pylorus/ or stomach antrum/ or stomach corpus/ or stomach epithelium/ or stomach fundus/ or stomach wall/) (5730)
14 (firmicute* or acidaminococc* or Bacteroidete* or Lactobacill* or lactobacteri* or lactococc* or bifidobacter* or streptococcus thermophilus or bacillus subtilis).tw,kf. (133273)
15 ((gastro* or gut* or gastric* or intestin* or stomach* or fecal* or faecal or f?ece* or stool or colon* or bowel* or ileum or ileal or duoden* or jejun* or digestive or cecum or sigmoid or splenic flexure or rectum or anal canal or ampulla of Vater or ileocecal valve or sphincter or cardia or forestomach or pylorus) adj6 (microb* or flora or microflor* or micro-flor* or microorgan* or micro-organ* or microecolog* or micro-ecolog* or dysbios* or dysbacterios* or disbacteri* or disbios* or symbio* or Actinobacteri* or Proteobacteri* or Escherichi* or Candida or Saccharomyces or Aspergillus or Penicillium or Rhodotorula or Trametes or Pleospora or Sclerotinia or Bullera or Galactomyces)).tw,kf. (158791)
16 ((f?ecal or stool or f?ece* or microb*) adj4 (transplant* or therap* or transfer* or infusion*)).tw,kf. (21259)
17 (gut adj2 brain).tw,kf. (11737)
18 enteric bacteria*.tw,kf. (4944)
19 prebiotic agent/ or probiotic agent/ (59780)
20 (probiotic* or prebiotic* or psychobiotic*).tw,kf. (59367)
21 or/9-20 (341968)
22 pediatrics/ or young adult/ or adolescent/ or child/ or preschool child/ or infant/ or baby/ or newborn/ or prematurity/ (4777204)
23 (infant* or baby or babies or newborn* or neonat* or minor or minors or toddler* or boy or boys or boyhood or girl or girls or girlhood or kid or kids or kiddie or child* or schoolchild* or adolescen* or preadolescen* or juvenil* or youth* or teen* or preteen* or underage* or under age* or pubescen* or prepubescen* or pre mature* or premature* or puberty or p?ediatric* or school* or schoolage*).tw,kf. (4602645)
24 (young* adj3 (adult* or person* or individual* or people* or population* or man or men or wom?n or male* or female*)).tw,kf. (469633)
25 (youngster* or preschool* or kindergar?en* or freshm?n* or junior* or soph?more* or senior* or highschool* or colleg* or universit* or student* or undergrad* or middleschool*).tw,kf. (1581398)
26 ((grade* or form*) adj3 (one or first or two or second or three or third or four* or five or fifth or six* or seven* or eight* or nine* or ninth or ten* or eleven* or twelfth or twelve or "1" or "2" or "3" or "4" or "5" or "6" or "7" or "8" or "9" or "10" or "11" or "12")).tw,kf. (864918)
27 all age*.tw,kf. (74314)
28 or/22-27 (8467013)
29 8 and 21 and 28 (5070)

**Interface - EBSCOhost Research Databases**
**Search Screen - Advanced Search**
**Database – CINAHL**

| # | Query | Results |
| --- | --- | --- |
| S1 | (MH "Depression") OR (MH "Depression, Postpartum") OR (MH "Depression, Reactive") OR (MH "Dysthymic Disorder") OR (MH "Premenstrual Dysphoric Disorder") OR (MH "Seasonal Affective Disorder") OR (MH "Mental Disorders") OR (MH "Affective Disorders") OR (MH "Mental Health") | 249,196 |
| S2 | TI ( depress* or dysthymia or sad or sadness or saddest or sadder or melanchol* or miser* or unhappiness or unhappy or emotion* or mood* or dysthymi* or MDD or affective* or dysphoric* or mental* or psychiat* or psychopath* or behavio?r* or anxiodepress* or sorrow* ) OR AB ( depress* or dysthymia or sad or sadness or saddest or sadder or melanchol* or miser* or unhappiness or unhappy or emotion* or mood* or dysthymi* or MDD or affective* or dysphoric* or mental* or psychiat* or psychopath* or behavio?r* or anxiodepress* or sorrow* ) | 630,531 |
| S3 | TI internal* OR AB internal* | 104,524 |
| S4 | (MH "Anxiety") OR (MH "Anticipatory Anxiety") OR (MH "Dental Anxiety") OR (MH "Separation Anxiety") OR (MH "Test Anxiety") OR (MH "Anxiety Disorders") | 73,668 |
| S5 | TI ( anxiet* or anxious* or hypervigilan* or nervous* or angst* or worry or worries or apprehensi* or uneasiness or unease or fear or fears or distress* or phobia* ) OR AB ( anxiet* or anxious* or hypervigilan* or nervous* or angst* or worry or worries or apprehensi* or uneasiness or unease or fear or fears or distress* or phobia* ) | 248,320 |
| S6 | S1 OR S2 OR S3 OR S4 OR S5 | 906,322 |
| S7 | (MH "Brain-Gut Axis") OR (MH "Gut Microbiota") OR (MH "Fecal Microbiota Transplantation") | 6,315 |
| S8 | (MH "Lactobacillus") | 3,092 |
| S9 | (MH "Microbiota") AND ((MH "Gastrointestinal System") OR (MH "Intestines") OR (MH "Intestine, Large") OR (MH "Colon") OR (MH "Sigmoid") OR (MH "Colon, Ascending") OR (MH "Cecum") OR (MH "Rectum") OR (MH "Intestine, Small") OR (MH "Duodenum") OR (MH "Anus") OR (MH "Jejunum") OR (MH "Ileum") OR (MH "Stomach")) | 684 |
| S10 | TI ( firmicute* or acidaminococc* or Bacteroidete* or Lactobacill* or lactobacteri* or lactococc* or bifidobacter* or streptococcus thermophilus or bacillus subtilis ) OR AB ( firmicute* or acidaminococc* or Bacteroidete* or Lactobacill* or lactobacteri* or lactococc* or bifidobacter* or streptococcus thermophilus or bacillus subtilis ) | 7,000 |
| S11 | TI ( (gastro* or gut* or gastric* or intestin* or stomach* or fecal* or faecal or f?ece* or stool or colon* or bowel* or ileum or ileal or duoden* or jejun* or digestive or cecum or sigmoid or splenic flexure or rectum or anal canal or ampulla of vater or ileocecal valve or sphincter or cardia or forestomach or pylorus) N6 (microb* or flora or microflor* or micro-flor* or microorgan* or micro-organ* or microecolog* or micro-ecolog* or dysbios* or dysbacterios* or disbacteri* or disbios* or symbio* or Actinobacteri* or Proteobacteri* or Escherichi* or Candida or Saccharomyces or Aspergillus or Penicillium or Rhodotorula or Trametes or Pleospora or Sclerotinia or Bullera or Galactomyces) ) OR AB ( (gastro* or gut* or gastric* or intestin* or stomach* or fecal* or faecal or f?ece* or stool or colon* or bowel* or ileum or ileal or duoden* or jejun* or digestive or cecum or sigmoid or splenic flexure or rectum or anal canal or ampulla of vater or ileocecal valve or sphincter or cardia or forestomach or pylorus) N6 (microb* or flora or microflor* or micro-flor* or microorgan* or micro-organ* or microecolog* or micro-ecolog* or dysbios* or dysbacterios* or disbacteri* or disbios* or symbio* or Actinobacteri* or Proteobacteri* or Escherichi* or Candida or Saccharomyces or Aspergillus or Penicillium or Rhodotorula or Trametes or Pleospora or Sclerotinia or Bullera or Galactomyces) ) | 16,810 |
| S12 | TI ( (f?ecal or stool or f?ece* or microb*) N4 (transplant* or therap* or transfer* or infusion*) ) OR AB ( (f?ecal or stool or f?ece* or microb*) N4 (transplant* or therap* or transfer* or infusion*) ) | 2,588 |
| S13 | TI (gut N2 brain) OR AB (gut N2 brain) | 1,402 |
| S14 | TI enteric bacteria* OR AB enteric bacteria* | 190 |
| S15 | (MH "Prebiotics") OR (MH "Probiotics") | 10,337 |
| S16 | TI ( probiotic* or prebiotic* or psychobiotic*) ) OR AB ( probiotic* or prebiotic* or psychobiotic* ) | 9,097 |
| S17 | S7 OR S8 OR S9 OR S10 OR S11 OR S12 OR S13 OR S14 OR S15 OR S16 | 31,756 |
| S18 | (MH "Pediatrics") | 21,412 |
| S19 | (MH "Young Adult") | 291,308 |
| S20 | (MH "Adolescence") | 608,572 |
| S21 | (MH "Child") | 528,760 |
| S22 | (MH "Child, Preschool") OR (MH "Schools") OR (MH "Colleges and Universities") OR (MH "Students") OR (MH "Minors (Legal)") | 289,677 |
| S23 | (MH "Infant") OR (MH "Infant, Newborn") OR (MH "Infant, Large for Gestational Age") OR (MH "Infant, Low Birth Weight") OR (MH "Infant, Postmature") OR (MH "Infant, Premature") | 289,102 |
| S24 | TI ( infant* or baby or babies or newborn* or neonat* or minor or minors or toddler* or boy or boys or boyhood or girl or girls or girlhood or kid or kids or kiddie or child* or schoolchild* or adolescen* or preadolescen* or juvenil* or youth* or teen* or preteen* or underage* or under age* or pubescen* or prepubescen* or pre mature* or premature* or puberty or p?ediatric* or school* or schoolage* ) OR AB ( infant* or baby or babies or newborn* or neonat* or minor or minors or toddler* or boy or boys or boyhood or girl or girls or girlhood or kid or kids or kiddie or child* or schoolchild* or adolescen* or preadolescen* or juvenil* or youth* or teen* or preteen* or underage* or under age* or pubescen* or prepubescen* or pre mature* or premature* or puberty or p?ediatric* or school* or schoolage* ) | 1,088,648 |
| S25 | TI ( young* N3 (adult* or person* or individual* or people* or population* or man or men or wom?n or male* or female*) ) OR AB ( young* N3 (adult* or person* or individual* or people* or population* or man or men or wom?n or male* or female*) ) | 128,269 |
| S26 | TI ( youngster* or preschool* or kindergar?en* or freshm?n* or junior* or soph?more* or senior* or highschool* or colleg* or universit* or student* or undergrad* or middleschool* ) OR AB ( youngster* or preschool* or kindergar?en* or freshm?n* or junior* or soph?more* or senior* or highschool* or colleg* or universit* or student* or undergrad* or middleschool* ) | 458,303 |
| S27 | TI ( (grade* or form*) N3 (one or first or two or second or three or third or four* or five or fifth or six* or seven* or eight* or nine* or ninth or ten* or eleven* or twelfth or twelve or "1" or "2" or "3" or "4" or "5" or "6" or "7" or "8" or "9" or "10" or "11" or "12") ) OR AB ( (grade* or form*) N3 (one or first or two or second or three or third or four* or five or fifth or six* or seven* or eight* or nine* or ninth or ten* or eleven* or twelfth or twelve or "1" or "2" or "3" or "4" or "5" or "6" or "7" or "8" or "9" or "10" or "11" or "12") ) | 88,228 |
| S28 | TI all age* OR AB all age* | 13,153 |
| S29 | S18 OR S19 OR S20 OR S21 OR S22 OR S23 OR S24 OR S25 OR S26 OR S27 OR S28 | 2,054,200 |
| S30 | S6 AND S17 AND S29 | 535 |

**Interface - EBSCOhost Research Databases**
**Search Screen - Advanced Search**
**Database - Child Development & Adolescent Studies**

| **#** | **Query** | **Results** |
| --- | --- | --- |
| S7 | S1 AND S6 | 30 |
| S6 | S2 OR S3 OR S4 OR S5 | Display |
| S5 | TI gut N2 brain OR AB gut N2 brain OR KW gut N2 brain | Display |
| S4 | TI ( ((f?ecal or stool or f?ece* or microb*) N4 (transplant* or therap* or transfer* or infusion*)) ) OR AB ( ((f?ecal or stool or f?ece* or microb*) N4 (transplant* or therap* or transfer* or infusion*)) ) OR KW ( ((f?ecal or stool or f?ece* or microb*) N4 (transplant* or therap* or transfer* or infusion*)) ) | Display |
| S3 | TI ( ((gastro* or gut* or gastric* or intestin* or stomach* or fecal* or faecal or f?ece* or stool or colon* or bowel* or ileum or ileal or duoden* or jejun* or digestive or cecum or sigmoid or splenic flexure or rectum or anal canal or ampulla of vater or ileocecal valve or sphincter or cardia or forestomach or pylorus) N6 (microb* or flora or microflor* or micro-flor* or microorgan* or micro-organ* or microecolog* or micro-ecolog* or dysbios* or dysbacterios* or disbacteri* or disbios* or symbio* or Actinobacteri* or Proteobacteri* or Escherichi* or Candida or Saccharomyces or Aspergillus or Penicillium or Rhodotorula or Trametes or Pleospora or Sclerotinia or Bullera or Galactomyces)) ) OR AB ( ((gastro* or gut* or gastric* or intestin* or stomach* or fecal* or faecal or f?ece* or stool or colon* or bowel* or ileum or ileal or duoden* or jejun* or digestive or cecum or sigmoid or splenic flexure or rectum or anal canal or ampulla of vater or ileocecal valve or sphincter or cardia or forestomach or pylorus) N6 (microb* or flora or microflor* or micro-flor* or microorgan* or micro-organ* or microecolog* or micro-ecolog* or dysbios* or dysbacterios* or disbacteri* or disbios* or symbio* or Actinobacteri* or Proteobacteri* or Escherichi* or Candida or Saccharomyces or Aspergillus or Penicillium or Rhodotorula or Trametes or Pleospora or Sclerotinia or Bullera or Galactomyces)) ) OR KW ( ((gastro* or gut* or gastric* or intestin* or stomach* or fecal* or faecal or f?ece* or stool or colon* or bowel* or ileum or ileal or duoden* or jejun* or digestive or cecum or sigmoid or splenic flexure or rectum or anal canal or ampulla of vater or ileocecal valve or sphincter or cardia or forestomach or pylorus) N6 (microb* or flora or microflor* or micro-flor* or microorgan* or micro-organ* or microecolog* or micro-ecolog* or dysbios* or dysbacterios* or disbacteri* or disbios* or symbio* or Actinobacteri* or Proteobacteri* or Escherichi* or Candida or Saccharomyces or Aspergillus or Penicillium or Rhodotorula or Trametes or Pleospora or Sclerotinia or Bullera or Galactomyces)) ) | Display |
| S2 | TI ( (firmicute* or acidaminococc* or Bacteroidete* or Lactobacill* or lactobacteri* or lactococc* or bifidobacter* or streptococcus thermophilus or bacillus subtilis or enteric bacteria* or probiotic* or prebiotic* or psychobiotic*) ) OR AB ( (firmicute* or acidaminococc* or Bacteroidete* or Lactobacill* or lactobacteri* or lactococc* or bifidobacter* or streptococcus thermophilus or bacillus subtilis or enteric bacteria* or probiotic* or prebiotic* or psychobiotic*) ) OR KW ( (firmicute* or acidaminococc* or Bacteroidete* or Lactobacill* or lactobacteri* or lactococc* or bifidobacter* or streptococcus thermophilus or bacillus subtilis or enteric bacteria* or probiotic* or prebiotic* or psychobiotic*) ) | Display |
| S1 | TI ( (depress* or dysthymia or sad or sadness or saddest or sadder or melanchol* or miser* or unhappiness or unhappy or emotion* or mood* or dysthymi* or MDD or affective* or dysphoric* or mental* or psychiat* or psychopath* or behavio?r* or anxiodepress* or sorrow* or internal* or anxiet* or anxious* or hypervigilan* or nervous* or angst* or worry or worries or apprehensi* or uneasiness or unease or fear or fears or distress* or phobia*) ) OR AB ( (depress* or dysthymia or sad or sadness or saddest or sadder or melanchol* or miser* or unhappiness or unhappy or emotion* or mood* or dysthymi* or MDD or affective* or dysphoric* or mental* or psychiat* or psychopath* or behavio?r* or anxiodepress* or sorrow* or internal* or anxiet* or anxious* or hypervigilan* or nervous* or angst* or worry or worries or apprehensi* or uneasiness or unease or fear or fears or distress* or phobia*) ) OR KW ( (depress* or dysthymia or sad or sadness or saddest or sadder or melanchol* or miser* or unhappiness or unhappy or emotion* or mood* or dysthymi* or MDD or affective* or dysphoric* or mental* or psychiat* or psychopath* or behavio?r* or anxiodepress* or sorrow* or internal* or anxiet* or anxious* or hypervigilan* or nervous* or angst* or worry or worries or apprehensi* or uneasiness or unease or fear or fears or distress* or phobia*) ) | Display |

**Database: APA PsycInfo <1806 to October Week 4 2023>**
**Search Strategy:**
**1** major depression/ or anaclitic depression/ or dysthymic disorder/ or endogenous depression/ or reactive depression/ or recurrent depression/ or treatment resistant depression/ or atypical depression/ or "depression (emotion)"/ or internalizing symptoms/ or persistent depressive disorder/ or seasonal affective disorder/ (184924)
2 mental health/ or youth mental health/ (92991)
3 anxiety disorders/ or generalized anxiety disorder/ or anxiety/ or death anxiety/ or illness anxiety disorder/ or performance anxiety/ or social anxiety/ or speech anxiety/ or test anxiety/ or travel anxiety/ (111279)
4 mental disorders/ (97533)
5 (depress* or dysthymia or sad or sadness or saddest or sadder or melanchol* or miser* or unhappiness or unhappy or emotion* or mood* or dysthymi* or MDD or affective* or dysphoric* or mental* or psychiat* or psychopath* or behavio?r* or anxiodepress* or sorrow* or internal*).ti,ab. (2164316)
6 (anxiet* or anxious* or hypervigilan* or nervous* or angst* or worry or worries or apprehensi* or uneasiness or unease or fear or fears or distress* or phobia*).ti,ab. (451739)
7 phobias/ (5834)
8 1 or 2 or 3 or 4 or 5 or 6 or 7 (2331602)
9 gastrointestinal microbiota/ (578)
10 microorganisms/ and (gastrointestinal system/ or intestines/ or stomach/) (775)
11 (firmicute* or acidaminococc* or Bacteroidete* or Lactobacill* or lactobacteri* or lactococc* or bifidobacter* or streptococcus thermophilus or bacillus subtilis or enteric bacteria* or probiotic* or prebiotic* or psychobiotic*).ti,ab. (965)
12 ((gastro* or gut* or gastric* or intestin* or stomach* or fecal* or faecal or f?ece* or stool or colon* or bowel* or ileum or ileal or duoden* or jejun* or digestive or cecum or sigmoid or splenic flexure or rectum or anal canal or ampulla of vater or ileocecal valve or sphincter or cardia or forestomach or pylorus) adj6 (microb* or flora or microflor* or micro-flor* or microorgan* or micro-organ* or microecolog* or micro-ecolog* or dysbios* or dysbacterios* or disbacteri* or disbios* or symbio* or Actinobacteri* or Proteobacteri* or Escherichi* or Candida or Saccharomyces or Aspergillus or Penicillium or Rhodotorula or Trametes or Pleospora or Sclerotinia or Bullera or Galactomyces)).ti,ab. (2252)
13 ((f?ecal or stool or f?ece* or microb*) adj4 (transplant* or therap* or transfer* or infusion*)).ti,ab. (276)
14 (gut adj2 brain).ti,ab. (1268)
15 9 or 10 or 11 or 12 or 13 or 14 (3140)
16 pediatrics/ (31998)
17 students/ or college students/ or elementary school students/ or high school graduates/ or high school students/ or junior high school students/ or kindergarten students/ or middle school students/ or preschool students/ (233099)
18 (infant* or baby or babies or newborn* or neonat* or minor or minors or toddler* or boy or boys or boyhood or girl or girls or girlhood or kid or kids or kiddie or child* or schoolchild* or adolescen* or preadolescen* or juvenil* or youth* or teen* or preteen* or underage* or under age* or pubescen* or prepubescen* or pre mature* or premature* or puberty or p?ediatric* or school* or schoolage* or youngster* or preschool* or kindergar?en* or freshm?n* or junior* or soph?more* or senior* or highschool* or colleg* or universit* or student* or undergrad* or middleschool* or all age*).ti,ab. (1871189)
19 (young* adj3 (adult* or person* or individual* or people* or population* or man or men or wom?n or male* or female*)).ti,ab. (148056)
20 ((grade* or form*) adj3 (one or first or two or second or three or third or four* or five or fifth or six* or seven* or eight* or nine* or ninth or ten* or eleven* or twelfth or twelve or "1" or "2" or "3" or "4" or "5" or "6" or "7" or "8" or "9" or "10" or "11" or "12")).ti,ab. (140537)
21 16 or 17 or 18 or 19 or 20 (1991808)
22 8 and 15 and 21 (394)

**Elsevier Scopus**

( ( TITLE-ABS-KEY ( ( young* W/3 ( adult* OR person* OR individual* OR people* OR population* OR man OR men OR wom?n OR male* OR female* ) ) ) ) OR ( TITLE-ABS-KEY ( ( youngster* OR preschool* OR kindergar?en* OR freshm?n* OR junior* OR soph?more* OR senior* OR highschool* OR colleg* OR universit* OR student* OR undergrad* OR middleschool* ) ) ) OR ( TITLE-ABS-KEY ( ( ( grade* OR form* ) W/3 ( one OR first OR two OR second OR three OR third OR four* OR five OR fifth OR six* OR seven* OR eight* OR nine* OR ninth OR ten* OR eleven* OR twelfth OR twelve OR "1" OR "2" OR "3" OR "4" OR "5" OR "6" OR "7" OR "8" OR "9" OR "10" OR "11" OR "12" ) ) . ) ) OR ( TITLE-ABS-KEY ( ( infant* OR baby OR babies OR newborn* OR neonat* OR minor OR minors OR toddler* OR boy OR boys OR boyhood OR girl OR girls OR girlhood OR kid OR kids OR kiddie OR child* OR schoolchild* OR adolescen* OR preadolescen* OR juvenil* OR youth* OR teen* OR preteen* OR underage* OR "under age*" OR pubescen* OR prepubescen* OR "pre mature*" OR premature* OR puberty OR p?ediatric* OR school* OR schoolage* OR all AND age* ) ) ) ) AND ( ( TITLE-ABS-KEY ( ( ( gastro* OR gut* OR gastric* OR intestin* OR stomach* OR fecal* OR faecal OR f?ece* OR stool OR colon* OR bowel* OR ileum OR ileal OR duoden* OR jejun* OR digestive OR cecum OR sigmoid OR "splenic flexure" OR rectum OR "anal canal" OR "ampulla of vater" OR "ileocecal valve" OR sphincter OR cardia OR forestomach OR pylorus ) W/6 ( microb* OR flora OR microflor* OR micro-flor* OR microorgan* OR micro-organ* OR microecolog* OR micro-ecolog* OR dysbios* OR dysbacterios* OR disbacteri* OR disbios* OR symbio* OR actinobacteri* OR proteobacteri* OR escherichi* OR candida OR saccharomyces OR aspergillus OR penicillium OR rhodotorula OR trametes OR pleospora OR sclerotinia OR bullera OR galactomyces ) ) ) ) OR ( TITLE-ABS-KEY ( ( ( f?ecal OR stool OR f?ece* OR microb* ) W/4 ( transplant* OR therap* OR transfer* OR infusion* ) ) ) ) OR ( TITLE-ABS-KEY ( ( gut W/2 brain ) ) ) OR ( TITLE-ABS-KEY ( ( firmicute* OR acidaminococc* OR bacteroidete* OR lactobacill* OR lactobacteri* OR lactococc* OR bifidobacter* OR "streptococcus thermophilus" OR "bacillus subtilis" OR "enteric bacteri*" OR probiotic* OR prebiotic* OR psychobiotic* ) ) ) ) AND ( TITLE-ABS-KEY ( ( depress* OR dysthymia OR sad OR sadness OR saddest OR sadder OR melanchol* OR miser* OR unhappiness OR unhappy OR emotion* OR mood* OR dysthymi* OR mdd OR affective* OR dysphoric* OR mental* OR psychiat* OR psychopath* OR behavio?r* OR anxiodepress* OR sorrow* OR internal* OR anxiet* OR anxious* OR hypervigilan* OR nervous* OR angst* OR worry OR worries OR apprehensi* OR uneasiness OR unease OR fear OR fears OR distress* OR phobia* ) ) )

**Wiley Cohrane**

**ID** **Search** **Hits**

#1 [mh ^depression] or [mh ^"depressive disorder"] or [mh ^"depression, postpartum"] or [mh ^"depressive disorder, major"] or [mh ^"depressive disorder, treatment-resistant"] or [mh ^"dysthymic disorder"] or [mh ^"premenstrual dysphoric disorder"] or [mh ^"seasonal affective disorder"] or [mh ^"mood disorders"] or [mh ^"mental health"] or [mh ^"mental disorders"] 37238

#2 (depress* or dysthymia or sad or sadness or saddest or sadder or melanchol* or miser* or unhappiness or unhappy or emotion* or mood* or dysthymi* or MDD or affective* or dysphoric* or mental* or psychiat* or psychopath* or behavio?r* or anxiodepress* or sorrow*):ti,ab,kw 310332

#3 internal*:ti,ab,kw 29078

#4 [mh ^"anxiety disorders"] or [mh ^"anxiety, separation"] or [mh ^"dental anxiety"] or [mh ^"performance anxiety"] or [mh ^"test anxiety"] 6038

#5 (anxiet* or anxious* or hypervigilan* or nervous* or angst* or worry or worries or apprehensi* or uneasiness or unease or fear or fears or distress* or phobia*):ti,ab,kw 133945

#6 {or #1-#5} 391346

#7 [mh ^"gastrointestinal microbiome"] or [mh ^"fecal microbiota transplantation"] or [mh ^"brain-gut axis"] 1471

#8 [mh ^firmicutes] or [mh ^acidaminococcus] or [mh ^Bacteroidetes] or [mh ^Lactobacillus] 1101

#9 [mh ^microbiota] and ([mh ^"gastrointestinal tract”] or [mh ^intestines] or [mh ^”intestine, large”] or [mh ^"anal canal"] or [mh ^cecum] or [mh ^colon] or [mh ^”colon, ascending”] or [mh ^”colon, descending”] or [mh ^”colon, sigmoid”] or [mh ^”colon, transverse”] or [mh ^rectum] or [mh ^”intestine, small”] or [mh ^duodenum] or [mh ^”ampulla of vater"] or [mh ^"sphincter of oddi"] or [mh ^ileum] or [mh ^jejunum] or [mh ^”lower gastrointestinal tract”] or [m

h ^stomach]) 67

#10 (firmicute* or acidaminococc* or Bacteroidete* or Lactobacill* or lactobacteri* or lactococc* or bifidobacter* or streptococcus thermophilus or bacillus subtilis):ti,ab,kw 9160

#11 ((gastro* or gut* or gastric* or intestin* or stomach* or fecal* or faecal or f?ece* or stool or colon* or bowel* or ileum or ileal or duoden* or jejun* or digestive or cecum or sigmoid or splenic flexure or rectum or anal canal or ampulla of vater or ileocecal valve or sphincter or cardia or forestomach or pylorus) near/6 (microb* or flora or microflor* or micro-flor* or microorgan* or micro-organ* or microecolog* or micro-ecolog* or dysbios* or dysbacterios* or disbacteri* or disbios* or symbio* or Actinobacteri* or Proteobacteri* or Escherichi* or Candida or Saccharomyces or Aspergillus or Penicillium or Rhodotorula or Trametes or Pleospora or Sclerotinia or Bullera or Galactomyces)):ti,ab,kw 25999

#12 ((f?ecal or stool or f?ece* or microb*) near/4 (transplant* or therap* or transfer* or infusion*)):ti,ab,kw 10688

#13 (gut near/2 brain):ti,ab,kw 573

#14 enteric bacteria*:ti,ab,kw 540

#15 [mh ^Prebiotics] or [mh ^Probiotics] 3235

#16 (probiotic* or prebiotic* or psychobiotic*):ti,ab,kw 10936

#17 {or #7-#16} 40048

#18 [mh ^pediatrics] 1071

#19 [mh ^"young adult"] 85087

#20 [mh ^adolescent] 126020

#21 [mh ^child] 70026

#22 [mh ^”child, preschool”] or [mh ^schools] or [mh ^universities] or [mh ^students] or [mh ^minors] 41862

#23 [mh ^infant] or [mh ^"infant, newborn"] or [mh ^”infant, large for gestational age”] or [mh ^”infant, low birth weight”] or [mh ^”infant, small for gestational age”] or [mh ^”infant, very low birth weight”] or [mh ^”infant, extremely low birth weight”] or [mh ^"infant, postmature"] or [mh ^”infant, premature”] or [mh ^”infant, extremely premature”] 42134

#24 (infant* or baby or babies or newborn* or neonat* or minor or minors or toddler* or boy or boys or boyhood or girl or girls or girlhood or kid or kids or kiddie or child* or schoolchild* or adolescen* or preadolescen* or juvenil* or youth* or teen* or preteen* or underage* or under age* or pubescen* or prepubescen* or pre mature* or premature* or puberty or p?ediatric* or school* or schoolage*):ti,ab,kw 465436

#25 (young* near/3 (adult* or person* or individual* or people* or population* or man or men or wom?n or male* or female*)):ti,ab,kw 119634

#26 (youngster* or preschool* or kindergar?en* or freshm?n* or junior* or soph?more* or senior* or highschool* or colleg* or universit* or student* or undergrad* or middleschool*):ti,ab,kw 172422

#27 ((grade* or form*) near/3 (one or first or two or second or three or third or four* or five or fifth or six* or seven* or eight* or nine* or ninth or ten* or eleven* or twelfth or twelve or "1" or "2" or "3" or "4" or "5" or "6" or "7" or "8" or "9" or "10" or "11" or "12")):ti,ab,kw 92882

#28 all age*:ti,ab,kw 299329

#29 {or #18-#28} 821540

#30 #6 and #17 and #29 2012

**Supplementary Table 2.** Joanna Briggs Institute (JBI) Critical Appraisal for Case-Control Studies

| Author, Year | Cai, 2022 | Hao, 2023 | Ling, 2022 | Zhou, 2022 |
| --- | --- | --- | --- | --- |
| Q1. Were the groups comparable other than the presence of disease in cases or the absence of disease in controls? | Yes | Yes | Yes | Yes |
| Q2. Were cases and controls matched appropriately? | Yes | Yes | Yes | Yes |
| Q3. Were the same criteria used for the identification of cases and controls? | Yes | Yes | Yes | Yes |
| Q4. Was exposure measured in a standard, valid and reliable way? | No | No | No | No |
| Q5. Was exposure measured in the same way for cases and controls? | Yes | Yes | Yes | Yes |
| Q6. Were confounding factors identified? | Yes | Yes | Yes | Yes |
| Q7. Were strategies to deal with confounding factors stated? | Yes | Yes | Yes | Yes |
| Q8. Were outcomes assessed in a standard, valid and reliable way for cases and controls? | Yes | Yes | Yes | Yes |
| Q9. Was the exposure period of interest long enough to be meaningful?* | Not Applicable | Not Applicable | Not Applicable | Not Applicable |
| Q10. Was appropriate statistical analysis used? | Yes | Yes | Yes | Yes |
| Total | 8/9 | 8/9 | 8/9 | 8/9 |

*If the question was not applicable, the total score was reduced by the number of non-applicable questions.

**Supplementary Table 3.** Joanna Briggs Institute (JBI) Critical Appraisal for Cross-Sectional Studies

| Author, Year | Kraaij, 2022 | Van de Wouw, 2022 |
| --- | --- | --- |
| Q1. Were the criteria for inclusion in the sample clearly defined? | Yes | Yes |
| Q2. Were the study subjects and the setting described in detail? | Yes | Yes |
| Q3. Was the exposure measured validly and reliably? | Yes | Yes |
| Q4. Were objective, standard criteria used for measurement of the condition? | Yes | Yes |
| Q5. Were confounding factors identified? | Yes | Yes |
| Q6. Were strategies to deal with confounding factors stated? | Yes | Yes |
| Q7. Were the outcomes measured validly and reliably? | Yes | Yes |
| Q8. Was appropriate statistical analysis used? | Yes | Yes |
| Total | 8/8 | 8/8 |

**Supplementary Table 4.** Joanna Briggs Institute (JBI) Critical Appraisal for Cohort Studies

| Author, Year | Laue, 2022 | Ou, 2023 |
| --- | --- | --- |
| Q1. Were the two groups similar and recruited from the same population? | Yes | Yes |
| Q2. Were the exposures measured similarly to assign people to both exposed and unexposed groups? | Yes | Yes |
| Q3. Was the exposure measured validly and reliably? | Yes | Yes |
| Q4. Were confounding factors identified? | Yes | Yes |
| Q5. Were strategies to deal with confounding factors stated? | Yes | Yes |
| Q6. Were the groups/participants free of the outcome at the start of the study (or at the moment of exposure)?* | Not Applicable | Not Applicable |
| Q7. Were the outcomes measured validly and reliably? | Yes | Yes |
| Q8. Was the follow-up time reported sufficient to be long enough for outcomes to occur? | No | Yes |
| Q9. Was the follow-up complete, and if not, were the reasons for the loss of follow-up described and explored? | No | Yes |
| Q10. Were strategies to address incomplete follow-up utilized? | Unclear | Unclear |
| Q11. Was appropriate statistical analysis used? | Yes | Yes |
| Total | 7/9 | 9/9 |

*If the question was not applicable, the total score was reduced by the number of non-applicable questions.

**Supplementary Table 5.** National Heart, Lung, Brain Institute (NHLBI) Quality Assessment for Before-After (Pre-Post) Studies with No Control Group

| Author, Year | Dong, 2021 |
| --- | --- |
| Q1: Was the study question or objective clearly stated? | Yes |
| Q2: Were eligibility/selection criteria for the study population prespecified and clearly described? | Yes |
| Q3. Were the participants in the study representative of those who would be eligible for the test/service/intervention in the general or clinical population of interest? | No |
| Q4. Were all eligible participants that met the prespecified entry criteria enrolled? | Not reported |
| Q5. Was the sample size sufficiently large to provide confidence in the findings? | No |
| Q6. Was the test/service/intervention clearly described and delivered consistently across the study population? | Yes |
| Q7. Were the outcome measures prespecified, clearly defined, valid, reliable, and assessed consistently across all study participants? | Yes |
| Q8 Were the people assessing the outcomes blinded to the participants' exposures/interventions? | No |
| Q9. Was the loss to follow-up after baseline 20% or less? Were those lost to follow-up accounted for in the analysis? | Yes |
| Q10. Did the statistical methods examine changes in outcome measures from before to after the intervention? Were statistical tests done that provided p values for the pre-to-post changes? | Yes |
| Q11. Were outcome measures of interest taken multiple times before the intervention and multiple times after the intervention (i.e., did they use an interrupted time-series design)? | No |
| Q12.* If the intervention was conducted at a group level (e.g., a whole hospital, a community, etc.) did the statistical analysis take into account the use of individual-level data to determine effects at the group level? | Not Applicable |
| Total | 6/11 |

*If the question was not applicable, the total score was reduced by the number of non-applicable questions. Risk of bias rating (Low (75-100%), Moderate (25-75%), or High (0-25%)
